# Insights into effective rest breaks for reducing cognitive and musculoskeletal strain in farm machinery operators: a qualitative reflexive analysis

**DOI:** 10.1080/07853890.2025.2582067

**Published:** 2025-11-02

**Authors:** Udoka Okpalauwaekwe, Wadena D. Burnett, Stephan Milosavljevic

**Affiliations:** a Department of Family Medicine, University of Saskatchewan, Saskatoon, SK, Canada; b School of Rehabilitation Science, University of Saskatchewan, Saskatoon, SK, Canada; c Prairie Agricultural Machinery Institute, Humboldt, SK, Canada

**Keywords:** Whole-body vibration, occupational exposure, rest breaks, occupation health, ergonomics, qualitative research

## Abstract

**Background:**

Agricultural machinery operators are frequently exposed to whole-body vibration (WBV), which contributes to musculoskeletal discomfort, cognitive fatigue, and impaired balance. While engineering controls such as improved seating and suspension systems have been widely studied, there is growing interest in complementary behavioral interventions, such as regular movement-based rest breaks, to mitigate the negative health effects of WBV exposure.

**Objective:**

We explored operators’ perceptions of rest break activities aimed at reducing WBV-related strain, with attention to factors influencing uptake, acceptability, and real-world feasibility.

**Methods:**

This was a reflexive-descriptive qualitative piece, to a broader experimental study involving 15 participants (10 in-lab, 5 in-field). In-lab participants completed a WBV simulation protocol and evaluated structured break activities (sitting, walking, stretching); in-field participants were observed on machinery and interviewed about their usual practices. Thematic analysis was conducted using an inductive approach.

**Results:**

Five overarching themes emerged. Participants preferred movement-based breaks but noted barriers such as time constraints and ingrained work habits posed significant barriers to regular break-taking. Greater awareness of WBV’s long-term health impacts was considered as motivators. Perceptions of WBV exposure differed between lab and field participants, influencing the perceived urgency for rest breaks. While engineering controls (e.g. seat design) were valued, they were viewed as necessary but insufficient without complementary active self-care strategies.

**Conclusion:**

Movement-based breaks were perceived as beneficial, but their adoption requires flexible, context-sensitive integration into daily routines. Interventions like gaze stabilization exercises offer physiological benefit, but must be adapted to respect farmers’ work routines and productivity imperatives for successful uptake.

## KEY MESSAGES

1. Farm machinery operators favor active breaks (e.g., walking, stretching) over passive ones (e.g., sitting), citing benefits for both physical relief and mental clarity.
2. Despite recognizing the benefits of taking occasional breaks between vibration exposure, deeply ingrained work habits and time-sensitive agricultural demands (e.g., during harvest) pose significant barriers to regular break-taking.
3. Farm machinery operators are more receptive to ‘incidental’ or ‘dual-purpose’ movement (e.g., walking while inspecting equipment) than to rigidly scheduled breaks. Flexible and task-compatible strategies are preferred.
4. For uptake in agricultural settings, break interventions should respect the local contexts and realities of seasonal work intensity, cultural norms, and workflow interruptions.

## Introduction

Agricultural, and industrial machinery operators are frequently exposed to whole-body vibration (WBV) due to prolonged periods spent operating heavy machinery in challenging field conditions [1,2]. This exposure is a known risk factor for various adverse health outcomes, including musculoskeletal discomfort, impaired balance, and cognitive fatigue [3–5], which collectively impact both the short- and long-term well-being of operators. Prolonged WBV exposure, especially while seated, compounds the strain on the lower back and axial skeleton, leading to cumulative effects that can manifest as low back pain, reduced reaction time, and diminished cognitive performance [1,3,6]. This can, in turn, increase the likelihood of on-the-job injuries, particularly during periods when agricultural operators may experience greater physical or cognitive demands, such as during planting or harvest seasons [6,7]. Traditional approaches to managing WBV have primarily focused on engineering controls, such as adjustable seating [8–10], and improved suspension systems [10–12], which aim to absorb vibration and reduce its transmission to the operator. However, these solutions often fall short in providing sufficient relief, as they cannot fully eliminate vibration effects in off-road environments [8,11]. Operating agricultural machinery is often classified as sedentary work due to low metabolic equivalents (METs), yet it is biomechanically taxing owing to prolonged static postures and exposure to whole-body vibration (WBV). This dual burden (i.e. low energy expenditure coupled with high musculoskeletal strain) has been linked to elevated risks of fatigue, discomfort, and chronic injury. Recent occupational health guidance recommends integrating short, active breaks in such contexts to mitigate cumulative biomechanical load and preserve function during extended work shifts [3–5,13–15]. As a result, there has been growing interest in exploring alternative interventions, such as the incorporation of regular, short-duration rest breaks that include physical activities like stretching or walking [3,7,16]. Recent studies have shown that rest breaks could alleviate discomfort, help restore cognitive function, and mitigate the negative impacts of WBV exposure on reaction time and balance [3,7].

In this study, we examine agricultural machinery operators’ perceptions of activity breaks (rest breaks) to identify feasible and effective strategies for enhancing the uptake of rest break activities in the agricultural setting. Given the variability of field conditions, task demands, and time pressures faced by Canadian farmers and farm machinery operators, we also assess the practical challenges associated with adopting structured breaks, as well as preferences for break types that could be seamlessly integrated into daily work routines. This qualitative inquiry serves as a pilot and feasibility study to explore the contextual uptake and perceived value of rest break interventions among a small and heterogeneous sample of agricultural machinery operators.

## Methods

### Study design

This qualitative piece was part of a larger multiple methods study investigation exploring interventions to mitigate the effects of whole-body vibration (WBV) in agricultural machinery operators [7]. In the experimental component of the larger study, we had employed a repeated-measures design conducted over four laboratory sessions separated by at least seven days to reduce carry-over effects. Each session involved 1 h of seated whole-body vibration (WBV) exposure on a six-degree-of-freedom robotic platform (R3000 Parallel Robotic Rotopod, Vancouver, BC) programmed to replicate vibration profiles of agricultural tractors according to ISO 2631-1 field measurements. A tri-axial accelerometer was used to calibrate the platform’s vibration output within ±5% of field-observed tractor values. Following exposure, participants completed one of four randomized 5-minute intervention activities: (1) seated rest, (2) walking, (3) gaze stabilization with trunk mobility, or (4) gaze stabilization with walking. Randomization of activity order across sessions was determined using a balanced block random number generator to minimize allocation bias. Outcome measures included ratings of perceived discomfort (Borg CR10 scale), static balance and postural sway (20-second trials on a force plate sampled at 1000 Hz with a fourth-order Butterworth filter), and reaction time performance (Psychomotor Vigilance Task, NASA-PVT+ application on an iPod) [7]. To control unwanted variability, participants were instructed to avoid significant WBV exposure (e.g. operating heavy machinery) for 24 h before each session, and testing protocols were standardized across sessions. The methods and findings of this experimental part has been published.

In this present piece, we used a descriptive qualitative design to explore operators’ experiences, preferences, and perceptions regarding rest break activities across two settings viz: a controlled laboratory environment and real-world in-field contexts. As such, this study was framed as a feasibility and exploratory pilot due to its small sample size, diverse participant roles, and applied focus on identifying context-specific insights rather than generalizable outcomes.

### Interpretative lens

To guide our interpretation of participants’ perspectives, we employed the Health Belief Model (HBM) as a theoretical framework [17]. The HBM is a widely recognized model in health psychology and behavior change research that seeks to explain and predict individuals’ engagement (or lack thereof) in health-promoting behaviors [17]. It posits that an individual’s readiness to act is influenced by six core constructs: *(1) perceived susceptibility*: belief about the likelihood of experiencing a health issue, *(2) perceived severity*: belief about the seriousness of the consequences, *(3) perceived benefits*: belief in the efficacy of a recommended action, *(4) perceived barriers*: belief about the tangible and psychological costs of action, *(5) cues to action*: internal or external prompts that trigger behavior, and *(6) self-efficacy***:** confidence in one’s ability to take action) [17,18]. Given the applied and behavior-focused nature of our study (i.e. centered on the feasibility and perceived value of rest break interventions) we used the HBM to frame participants’ reflections on how and why they might adopt or resist short, active rest breaks. As such, this framework allowed us to examine how beliefs around health risks (e.g. WBV exposure), intervention effectiveness, perceived work constraints, and motivational cues influenced participants’ openness to integrating movement-based breaks into their routines.

### Setting and participants

Participants in this study were recruited both in-lab and in-field. Purposive sampling was used to capture variation in age, farming experience, and operational context. In-lab participants completed a WBV simulation protocol at the Ergonomics Laboratory of the University of Saskatchewan (Usask), undergoing one hour of seated vibration exposure on a simulated tractor platform, repeated four times over a seven-day period as part of a repeated measures experimental design [7]. Details of this repeated measures study have been previously published [7]. In-field participants were recruited from farms in Saskatchewan, Canada, and interviewed after their typical operational work routines. As a qualitative component of a broader multiple methods study, our sample size was guided by the principle of thematic saturation rather than statistical power, to which studies have shown that thematic (data) saturation can often be reached in qualitative studies where sample sizes are within the ranges of 9 to 17 with relatively homogenous populations and narrowly defined objectives [19,20].

### Data collection

Data for this study were collected between May 2022 and September 2023, encompassing both the controlled laboratory phase and in-field interviews with machinery operators. In the laboratory setting, participants completed structured break activities (sitting, walking, and stretching) following WBV exposure. They then participated in semi-structured interviews exploring their perceptions of each break type, comfort, and feasibility. In the field setting, participants were observed during routine machinery operation and subsequently completed interviews focused on their usual break practices, barriers to taking breaks, perceptions of WBV-related strain, and experiences with engineering controls such as seat comfort or suspension systems. All interviews were independently conducted by the Canadian Hub for Applied and Social Research (CHASR) at the University of Saskatchewan using an interview guide developed collaboratively by the research team and piloted with two operators. Interviews lasted 30–60 min, were audio-recorded with consent, and transcribed verbatim.

### Data analysis and trustworthiness

Transcripts were analyzed descriptively and thematically using Vears and Gillam’s processes for inductive coding [21], which includes: (1) read and familiarize with the data; (2) read through and develop the first set of ‘big-picture’ codes; (3) review and develop sub-categories of codes; (4) refine the sub-categories; and (5) synthesize codes into meaningful messaging with reflexive notes where necessary. Initial coding was conducted by UO following a first read and familiarization with the transcripts, after which iterative team discussions were held to develop a coding framework. Themes were derived inductively, and data were triangulated across in-lab and in-field contexts to identify both shared and divergent perspectives. Strategies to enhance rigor and trustworthiness included: code/concept triangulation, reflexive journaling, and participants’ validation of findings. While our coding and theme generation remained inductive, we subsequently applied constructs from the Health Belief Model (HBM) [17], to interpret participant responses related to perceived barriers, cues to action, and the feasibility of adopting rest break interventions in practice.

### Ethical considerations

All participants provided informed consent prior to participation in the study. Informed consent was obtained in writing from all participants before data collection commenced. No minors were included in this study. The study procedures were conducted in accordance with the Declaration of Helsinki and were approved by the University of Saskatchewan Biomedical Research Ethics Board (Bio-REB), Approval ID #2594 (March 21, 2022, updated through 2026). Consent to publish anonymized data excerpts, including participant quotes, was obtained in writing from all participants prior to study commencement. All efforts were made to de-identify participants, and no identifiable information is presented in this manuscript.

## Findings

We recruited a total of 15 participants (ten, in-lab and five, in-field). Our analysis revealed five overarching themes common to both in-lab and in-field interviews. We categorized these themes to reflect key discussion prompts in the interviews where we found commonalities between context in perspectives (i.e. in-lab vs in-field participants).

### Theme 1: ‘walking is good’–preferences for movement-based breaks

Both in-lab and in-field participants expressed clear preferences for breaks that involved physical movement, such as walking or stretching, over *passive* breaks such as sitting. Participants noted that movement-based breaks provided them both physical relief and a *mental* reset. They noted these active movement-based breaks were particularly useful in alleviating the fatigue and discomfort associated with long hours in the operator’s seat. The preference for movement suggests a desire for breaks that can actively counteract the physical strains of WBV exposure. See exemplar quote:

Walking was good and kind of stretching portion that went along with the gaze stabilization worked well. When you’ve been in a tractor for long periods, you can do some stretches instead of walking around for a bit.

Another in-lab participant elaborated on the restorative nature of walking:

I enjoyed the walking… it’s something that could be brainless as opposed to some of the trunk stretching. Sitting felt like it might contribute to body fatigue. Walking was the easiest one to convince a farmer to do.

Another inferred a dual benefit to walking rest breaks:

Walking around was just the most practical. It was easy to do, and you could be productive by checking on the equipment at the same time.

This reflection suggests a dual benefit of physical activity and task continuity.

Similarly, one in-field participant echoed the sentiment, stating:

If I need to go stretch, I’m gonna stop the tractor or walk around or use the tire to stretch my arms out. It helps me stay alert and keeps my back from getting too stiff

Here, we see a preference for movement-based breaks aligning with the participant’s need for control and flexibility in their routines, suggesting that effective break interventions could include short, movement-oriented activities that do not disrupt their workflow. Overall, movement during breaks under this theme was seen not only as a personal relief but as a dual-purpose action tied to ongoing task requirements, such as equipment checks or walking to other machinery.

#### Interpretation with HBM

This theme aligns with ‘*perceived benefits’* in the HBM [17,18], wherein participants identified walking and other movement-based breaks as producing tangible physical and cognitive relief. The integration of movement into ongoing tasks also suggests a higher likelihood of adoption, as perceived benefits outweigh perceived barriers in these contexts.

#### Reflection

It is important to note, participants in the lab experiments had the opportunity to try various structured break types, such as sitting, walking, and stretching, where they often noted specific preferences based on these structured experiences. This allowed them to identify rest or movement breaks they found most effective. As such, in-lab participants were able to make clearer preferences. For example one in-lab participant clarified: ‘I liked the walking best…it’s easy to integrate, and it gives a mental break too’. However, for participants in the field, breaks were generally incidental, often taken to perform other tasks like checking equipment or making repairs. Many in-field participants noted that breaks were taken on an *as-needed basis* rather than being planned, and they preferred integrating short movements into existing tasks rather than stopping entirely for a break. For example, one participant on the field noted: ‘When you’re already getting out to fix something, it’s easy to take a stretch, but I wouldn’t stop just to stretch’.

### Theme 2: ‘taking regular breaks is a bit of a stretch’–barriers to taking regular breaks

Like in many agricultural settings, there is the urgency associated with agricultural work. Timing can significantly impact outcomes, creating a practical barrier to taking structured breaks, especially when it meant stopping machinery or delaying a task. As such, time constraints were a recurring theme in this context. Time constraints, particularly during busy seasons like planting and harvest, were frequently cited as a considerable barrier to taking regular breaks. Both in-lab and in-field participants felt the pressure to maintain productivity, often foregoing breaks even when they felt a little discomfort from sitting for lengths in time in their machines. Several in-lab participants described the challenge of integrating breaks into the workday, saying,

It’s hard to stop when you’re in the middle of things. Even five minutes feels like too much sometimes, especially during critical times like harvest

The hurdle is going to be work habits. Nothing coming out of this study will override human nature. Farmers don’t take breaks unless they’re forced to.

Several in-field participants also expressed similar sentiments:

Stopping during harvest isn’t really an option; it has to be fast and practical, or it’s just not happening. Every minute counts, and it’s hard to justify taking a break

We get out to check sickles, but we don’t take a break just to stretch our legs. If we could fit it in while doing something else, maybe. But not for the sake of taking a break.

Again, these accounts underscore the salient challenge of taking rest-breaks by farmers/machine operators but also inform considerations for (co)developing break interventions that are short, flexible, and capable of being integrated into context-specific workflows of farmers/operators without significantly disrupting their productivity.

However, when discussing the possibility of adopting structured or timed rest breaks using reminder devices, participants expressed mixed views. In the lab setting, participants were generally more receptive to the concept of using reminder devices or apps to prompt breaks. They felt these tools could help establish a routine, particularly in controlled environments where reminders might not be as easily ignored. One participant suggested:

A reminder on the phone could be useful during long sessions, just to prompt you to get up and move a bit,

On the other hand, participants in the field were more skeptical of reminder devices, expressing concerns that reminders might be disruptive during busy times. Some noted that any such tool would need to be highly adaptable to their variable schedules and responsive to the dynamic nature of their tasks. One participant said:

If it goes off at the wrong time, it would just be an annoyance. We’re not sitting at a desk, so it needs to adapt to the work

#### Interpretation with HBM

This theme aligns with the ‘*perceived barriers*’ construct in the HBM [17,18]. Time pressures, task urgency, and workflow disruptions were significant deterrents to consistent break-taking, especially during high-stakes seasons; highlighting the importance of designing interventions that minimize perceived inconveniences and integrate into existing routines.

### Theme 3: ‘it’s necessary to improve our longevity’– the value of education and awareness of WBV impacts

Across both settings (in-lab and in-field), most participants expressed that greater awareness of the long-term health impacts of WBV exposure would encourage more consistent break-taking. Some participants noted that, understanding the cumulative effects of WBV on musculoskeletal health, cognitive fatigue, and reaction time might make it easier to prioritize regular breaks among the farming community. One participant shared:

Sometimes, you just don’t realize what these little breaks can do for you in the long run. Knowing it helps long-term would make it easier to stop for a few minutes.

Similarly, an in-field participant commented,

Sometimes you need a reminder that your health matters as much as getting things done quickly. A little education on how it helps would go a long way

Another participant linked health consciousness to both physical longevity and mental acuity:

I feel like my body wears the long-term ramifications of poor agricultural work habits. Any farmer older than I am would agree it’s necessary to improve our longevity.

Similar sentiments were echoed by an in-field participant:

Your own personal well-being is more important than the task. Just reminding people to take care of themselves…that’s what needs to be emphasized.

These responses suggest that educational efforts focused on the health benefits of breaks could enhance adoption, particularly if operators understood that short-term productivity sacrifices lead to long-term health gains.

#### Interpretation with HBM

This theme reflects both ‘*perceived severity*’ and ‘*cues to action*’ within the HBM [17,18]. Participants linked WBV exposure to long-term health decline and expressed that education could serve as a cue to change behavior. This could suggest that targeted awareness campaigns could enhance break-taking behaviors by enhancing perceived personal risk.

### Theme 4: ‘it’s not as rough as in the field’ – perceptions of WBV exposure across contexts

Participants’ motivation to adopt rest breaks may also be shaped by how seriously they perceive the severity of WBV exposure. Perceptions of WBV intensity varied between lab and field participants. In the lab setting, participants noted that simulated WBV exposure felt less intense than what they typically experience in the field, which may have influenced their experiences of the rest activities. One participant remarked:

It’s not as rough as what you’d feel in the field, but it still gives you an idea of what a break might feel like after sitting for a while.

Conversely, in the field, participants connected WBV exposure not only to personal discomfort but also to equipment wear and tear. They recognized that reducing WBV could benefit both their health and machinery performance, framing it as a dual incentive to adopt break practices:

Reducing vibration would help both me and the equipment. It’s not just about my back but also about keeping the machines running longer.

#### Interpretation with HBM

This theme further elaborates ‘*perceived susceptibility and severity*’ in the HBM [17,18]. Participants’ acknowledgment of WBV intensity (particularly in real-world conditions) indicates growing awareness of personal risk, which may shape motivation toward behavioral change.

### Theme 5. ‘…equipment makes a difference’– importance of engineering controls and seat comfort

Participants across both settings emphasized the importance of their equipment and design in relation to ergonomics (e.g. seat comfort and adjustability of controls). They believed that ergonomically designed equipment *could help* reduce the long-term effects of WBV but in itself is insufficient. For example, adjustable seating, lumbar support, and suspension systems were frequently mentioned as mitigating features, though cost was often a limiting factor. It is possible that the focus on seat comfort reflects a desire for solutions that can provide passive relief from WBV, complementing a need for regular activity breaks. See exemplar quotes:

An air-ride seat would have made a huge difference in comfort during the session. I could see that helping a lot in the field too.

Stretching is good, but the equipment makes a difference too. The smaller or older tractors are rougher. It’s not just your back-it’s the whole machine.

The bumps in a tractor can be pretty jarring, especially if you’re in there too long without ear protection.

Some in-field participants also noted,

I’ve put extra support in my seat, but I’m not sure it does enough to really reduce the vibration. Sometimes, just having something to lean on makes all the difference.

We don’t have time for walking breaks. We need something like better seats to take the edge off the vibration when we can’t stop.

#### Interpretation with HBM

This theme may be mapped to ‘*self-efficacy and modifying factors*’ in the HBM [17,18]. When participants highlighted equipment-based solutions, they were expressing both a need for systemic support and their confidence in managing discomfort through accessible means, which could either enhance or detract from break-taking behaviors.

## Discussion

This qualitative study builds upon our prior experimental research [7], by extending understanding of how rest break activities, particularly those involving gaze stabilization exercises (GSE) combined with active mobility (e.g. stretching, walking), might be feasibly adopted in real-world agricultural settings. While our experimental findings demonstrated that such interventions effectively preserved cognitive reaction time following simulated WBV exposure [7], participants’ narratives in this reflexive qualitative piece emphasized that physiological efficacy alone was insufficient to ensure in-field adoption or movement-based breaks. Rather, uptake appeared contingent on the pragmatic integration of movement-based breaks into farmers’ or machine operators’ existing work routines and effort requirements. Participants notably favored ‘incidental movements’ or ‘task-integrated movements’ (i.e. embedding or integrating mobility-based activities like stretches or brief walks within routine tasks, such as equipment inspections) over rigidly structured or timed interventions. When interpreted through the Health Belief Model [17,18], participants’ experiences reflected multiple interlocking constructs (ranging from perceived barriers to perceived severity and self-efficacy). These insights further offer a behavioral framework for designing interventions that not only educate but address context-specific constraints faced by machinery operators.

Thus, a successful adoption of movement-based (rest) breaks will likely depend on presenting rest break strategies as flexible, self-directed tools that align with the famers’ or machine operators’ workflow demands, rather than an externally imposed schedule. Our findings also underscore a broader behavioral distinction between *proactive* and *reactive* rest break behaviors. While occupational health literature tends to support *proactive, scheduled breaks* (i.e. taken before discomfort or fatigue arises) as more effective for injury prevention and performance preservation [14], many participants in this study reported taking breaks only *reactively*, when discomfort had already set in or when a task allowed it. This pattern aligns with elements of the Health Belief Model, in which *cues to action***,** *perceived susceptibility*, and *workflow fit* all influence whether a break is adopted. To improve uptake, interventions may need to incorporate proactive prompts (e.g. personalized reminders or built-in cues in farm machinery) that are flexible enough to adapt to unpredictable workflows yet still encourage early, preventive actions rather than reactive ones. How such a strategy can be made pragmatically feasible, compatible with everyday tasks, beneficial to health, and yet financially prudent remains a focal area for future inquiry. Such an investigation would likely use both quantitative and qualitative methodologies to provide a cost benefit analysis that would examine the financial implications of committing dedicated time to physiologically beneficial breaks from continued vibration exposure. Consistent with findings from the transportation and mining sectors [1–3,6,10,22], participants in our study valued physical movement as a strategy to alleviate WBV-induced discomfort and fatigue. However, our findings extend this literature by illuminating a distinct cultural and operational preference among Canadian farmers for dual-purpose breaks (i.e. those that simultaneously support both physical relief and ongoing task completion). This preference may reflect the distinctive operational pressures faced in Canadian agriculture, where short growing seasons, long winters, and challenging soil conditions necessitate maximizing productivity within narrow timeframes [7,23,24]. The tension between productivity demands and health-promoting behaviors was especially evident during peak agricultural seasons, echoing prior findings on barriers to preventive practices in farming [24]. Interestingly, while many interventional studies have focused heavily on engineering controls (e.g. seat suspension) [2,8,9,12,25], our participants consistently (across both in-lab and in-field settings) viewed such equipment enhancements as necessary but insufficient. This dual emphasis on passive (engineering) and active (behavioral) strategies suggests that future interventions should couple biomechanical supports with behaviorally acceptable formats to ensure both physiological effectiveness and practical adoption. To the best of our knowledge, very few studies [3,7], have explored how farm operators themselves perceive the feasibility of rest break interventions following WBV exposure. Our work addresses this gap by surfacing nuanced operator insights regarding barriers (e.g. time constraints, cultural norms), motivators (e.g. health longevity, injury prevention), and design preferences (e.g. flexibility, autonomy). Moreover, our findings build upon Burnett et al. (2023) by reinforcing that while structured activities like GSE with mobility can physiologically sustain reaction time, successful field adoption will likely depend on empowering the agricultural workforce to more fully understand the implications of this occupational risk; engage with their peers (and health advisors) to co-develop context-sensitive intervention strategies that align with the demands and dynamics of their agricultural work.

## Study limitations

While this study provides valuable insights into machinery operators’ perceptions of rest break interventions, several limitations warrant consideration. First, the WBV exposure simulated in the laboratory setting was noted by some participants to be less intense than real-world field exposures, which may have influenced their perceptions of rest break needs and effectiveness. Second, participants’ experiences differed between settings: in-lab participants evaluated structured rest activities (e.g. sitting, walking, stretching), whereas in-field participants described more opportunistic, task-integrated movements. These contextual differences may have shaped the nature and strength of participant preferences and limit the *direct* generalizability of our findings across settings. Future research should carefully tailor interventions to real-world operational constraints and consider strategies to bridge the gap between experimental effectiveness and field-based feasibility.

Finally, the small sample size and heterogeneous nature of participants (e.g. variation in age, machinery types, work contexts, and roles) limit the generalizability of the findings. While the aim was to gather rich, contextually grounded insights into feasibility and perceptions of rest breaks, the results should be interpreted as exploratory rather than conclusive. Larger studies with stratified sampling and diverse agricultural contexts would be needed to validate these findings and assess broader applicability.

## Conclusion

Our qualitative reflexive study offers nuanced insights into how farm machinery operators experience and respond to rest break interventions aimed at reducing the adverse effects of WBV. Operators consistently preferred movement-based breaks for physical and mental relief but identified significant barriers such as time pressure, ingrained work habits, and workflow demands that limit the adoption of structured breaks. While an experimental study confirmed the physiological effectiveness of interventions like GSE, this qualitative analysis underscored the role of context, culture, and workflow compatibility in shaping rest break adoption. Interpreted through the Health Belief Model, participants’ perspectives illuminated the behavioral dimensions of intervention uptake, including perceived severity of WBV impacts, perceived benefits and barriers to taking breaks, and the importance of self-efficacy and environmental cues. These findings highlight the value of using behavioral frameworks to guide future intervention design. Taken together, our findings highlight the need for integrated strategies that combine biomechanical interventions with operator-centered approaches, to ensure that co-designed interventions are practical, adaptable, and sustainable in the ever-changing demands of agricultural work.

## Data Availability

The datasets used and/or analysed during the current study are available from the corresponding author on reasonable request.
